# Relation of Birthweight and Ovarian and Uterine Size Prior to Menarche

**DOI:** 10.1007/s43032-020-00351-y

**Published:** 2020-10-14

**Authors:** Nadia Parisi, Alice Tassi, Valentina Capodicasa, Anjeza Xholli, Angelo Cagnacci

**Affiliations:** 1 Institute of Obstetrics and Gynecology, Azienda Sanitaria Universitaria Friuli Centrale “Santa Maria Della Misericordia”, University Hospital of Udine, Udine, Italy; 2 Department of Obstetrics and Gynecology, San Martino Hospital, Genoa, Italy; 3 Università degli Studi di Genova, Genoa, Italy

**Keywords:** Birthweight, Fetal growth, Uterus, Ovary, Reproduction, Small for gestational age

## Abstract

During pregnancy, supply of nutrients and exposure of the mother to environmental factors can influence fetus phenotype, possibly modifying growth of fetal tissues and organs. Few studies inconsistently reported that fetuses exposed to an insufficient energy supply, as those born small for gestational age, may have a reduced volume of uterus and ovaries. A retrospective analysis was performed on ultrasound data performed between 2012 and 2018 in 69 young premenarchal girls, 5 to 9 years of age, attending our endocrine–gynecologic clinic for a suspect of early puberty. Length of pregnancy and birthweight was also retrieved. When corrected for age, and presence of ovarian follicles, ovarian volume was positively (*R*^2^ = 0.210; *p* = 0.001) related to percentiles of birthweight (beta coefficient 0.012; 95% CI, 0.002–0.021). Similarly, uterine volume was positively (*R*^2^ = 0.237; *p* = 0.005) related to percentiles of birthweight (beta coefficient 0.067; 95% CI, 0.021–0.114). Ovarian (*p* = 0.034) and uterine (*p* = 0.014) volume was higher in the upper 3rd distribution of birthweight percentiles. In conclusion, development of ovarian and uterine volume increases progressively with the increase of birthweight percentiles. The data indicate an association between birthweight and the volume of uterus and ovary at 5–9 years of age.

## Background

Pregnancy is a critical period for fetal phenotype characterization. Life in utero can permanently change body’s structure, function, and metabolism. Clear associations were observed between a low birthweight and cardiovascular disease, kidney disease, diabetes, and other sicknesses of adulthood [1].

Functional consequences on reproduction were also suggested. Fetuses born small for gestational age (SGA) had more elevated levels of gonadotrophins and insulin [2], an earlier menarche [3–6], reduced reproductive capacity [7], and a lower rate of polycystic ovary morphology in post-precocious pubarche women [8].

Growth and development of internal organs are influenced by intrauterine fetal growth. For example, in comparison with normal weight, SGA newborns have a reduced number of nephrons [9]. Internal genitalia share with kidneys the same embryological origin, but whether their development is conditioned by intrauterine fetal growth is still unclear. A reduced ovarian fraction of primordial follicles was found in four extremely retarded fetuses born at different gestational age [10], but this was not confirmed in a subsequent study [11]. Reduced ovarian and uterine volume at adolescence was reported in females born SGA [12], but in another study, intrauterine fetal growth, evaluated by ultrasonography, was not related to post-menarchal ovarian and uterine volume [13]. To further evaluate this issue, we evaluated whether dimension of internal genitalia of premenarchal girls is related to neonatal birthweight.

## Materials and Methods

Data were retrospectively retrieved by a database on ultrasounds performed at the University Hospital of Udine between 2012 and 2018. A total of 75 ultrasounds were performed in premenarchal girls 5 to 9 years of age, who attended our endocrine–gynecologic clinic for a suspect of premature thelarche, pubarche, or precocious puberty. Data anonymously retrieved were age at time of the ultrasound investigation, clinical reason for the examination, use of therapies, particularly hormonal therapies, weight at birth, and weeks of gestation at birth. Premature thelarche was defined as isolated unilateral or bilateral breast development without the development of other sexual characteristics [14]. Premature pubarche was defined as the onset of sexual hair at less than 8 years, with clinical and laboratory data excluding other pathologies and a sufficient follow-up to exclude true precocious puberty [14]. True precocious puberty was diagnosed in girls less than 8 years when pubarche was associated with an increased growth velocity, advanced skeletal maturation, increased LH, and LH response to GnRH [14]. Patients born with evidence of fetal growth restriction due to maternal hypothyroidism or chromosomal, syndromic, or infectious causes were excluded [15]. Girls lacking complete measurement of both ovaries and uterus were excluded. Final evaluations were performed in 69 girls. The retrieved data on birthweight were expressed as percentiles for gestational age, in accordance with prenatal curves [16]. SGA was defined as a female born below the 10th birthweight percentile.

Ultrasound scans had been performed by a transabdominal probe in a full-bladder state by two experienced sonographers by using a Voluson E8 equipped with a 2–8 MHz RAB4–8-D convex transducer (GE Medical Systems, Austria).

Measurements of the uterus were taken in longitudinal, transverse, and antero-posterior views. The same was made for ovary measurement. In several cases, ovarian parenchyma showed anechoic areas traceable to follicles. Presence of follicles was recorded. Ovarian and uterine volume was calculated using the formula for a modified prolate ellipsoid (longitudinal x transverse x antero-posterior *0.5233). The mean values of the volume of both ovaries were entered in statistical analysis.

The sample size was based on a previous study where 36 girls were sufficient to document a difference of uterine (− 20%) and ovarian (− 40%) volume between neonates born SGA and appropriate for gestational age (AGA) [12]. By placing a type I error at 5% and type 2 at 20%, 33 subjects were sufficient to document a uterine or ovarian volume difference of 30% between two groups. Descriptive statistic was used. Results for continuous data are expressed as mean + standard deviation, and for nominal data as median with interquartile range. The Mann–Whitney test and the Kruskal–Wallis test were used for comparing data of two or more groups, respectively. Contingency tables with the chi-squared test were used to compare frequencies. Simple linear regression analysis was used to evaluate the relation of either ovarian or uterine volume and independent variables. Independent variables were birthweight, expressed in percentiles at week of delivery, weeks at which the girl was delivered, and actual age in months. Nominal variables, such as presence of ovarian follicles vs. absence, and precocious puberty or premature pubarche vs. premature thelarche were entered as dummy variables. The relation of uterine volume with ovarian volume was also tested. Beta coefficient of linear regression with 95% confidence interval (95% CI) was reported for each analysis. For each regression model, the *R*^2^ is provided as a measure of the quality of the model. *R*^2^ indicates the proportion of the dependent variable’s variability that is explained by the independent variables. Multiple regression models were constructed entering variables significantly related to either ovarian or uterine volume. Variables not significantly related were not entered into the models. In all analyses, a *p* value < 0.05 was considered significant. Statistical analysis was performed using the statistical package StatView 5.01 (SAS Institute Inc., Cary, NC, USA).

## Results

Girls under study had a mean age of 7.7 ± 1.08 years (92.3 ± 13.2 months of age) and were born at a mean gestational age of 39.1 ± 2.2 weeks. Average birthweight was 3092 ± 519 g, and average of birthweight percentile was 30.2 ± 27.3. All girls were premenarchal. Twenty-nine had premature thelarche, 18 premature pubarche, and 22 precocious puberty. Of the latter, 6 were under GnRH analogs.

At ultrasound, median ovarian volume was 0.844 cm^3^ (0.537–1.489 cm^3^). Ovarian follicles were present in 46 cases. Median uterine volume was 1.450 cm^3^ (0.831–2.574 cm^3^).

Ovarian volume tended to be higher but not significantly so (*p* = 0.164) in girls with precocious puberty than premature thelarche or premature pubarche (Table 1). Uterine volume was similar in the 3 groups of women (Table 1). Similarly, week at birth or percentage of girls born small for gestational age was similar in the 3 groups of girls (Table 1). In girls born SGA (33.3%), volume of ovary (1.036 cm^3^ (0.397–1.675 cm^3^) vs. 0.837 cm^3^ (0.114–1.56 cm^3^); *p* = 0.741) and uterus (1.347 cm^3^ (0.134–2.560 cm^3^) vs. 1.570 cm^3^ (0.111–3.029 cm^3^); *p* = 0.582) was not significantly different from that of girls born AGA.

**Table 1:** Median and interquartile range of ovarian and uterine volume, week of birth, and percentage of girls born small for gestational age (SGA) among girls with premature thelarche (*n* = 29), pubarche (*n* = 18), or precocious puberty (*n* = 22)

	Premature thelarche	Premature pubarche	Precocious puberty	*p* value	Total
Ovary volume (cm^3^)	0.782 0.384–1.495	0.825 0.564–1.168	1.089 0.713–2.415	0.160	0.844 0.531–1.489
Uterus volume (cm^3^)	1.597 0.830–2.022	1.246 0.852–4.989	1.424 0.748–2.813	0.999	1.450 0.831–2.574
Week of birth	39 37–41	39 38–40	39 38–40	0.999	39 37.75–40.25
SGA	26.9%	44.4%	31.8%	0.271	33.3%

**Fig. 1:**
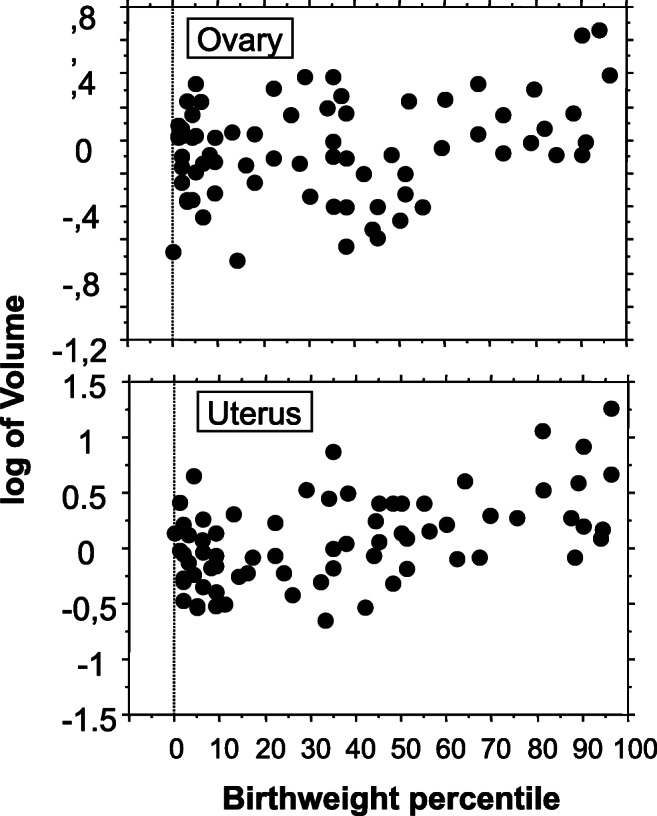
Scatterplot of log-transformed ovarian (top) and uterus (bottom) volume stratified by birthweight percentiles

Stratification of ovarian volume by percentiles of birthweight showed that ovarian volume was higher (*p* = 0.034) in girls with a birthweight above the 66th percentile (Table 2). Figure 1 shows the scatterplot of data that were log-transformed to reduce their dispersion. Stratification of uterine volume by percentiles of birthweight showed that uterine volume was higher (*p* = 0.014) in girls with a birthweight above the 66th percentile than in those with a birthweight between the 33rd and 66th percentiles or below it (Table 2). Figure 1 shows the scatterplot of log-transformed data.

**Table 2:** Median and inter-quartile range of ovarian and uterine volume, week of birth, and percentage of girls suffering from premature thelarche, premature pubarche, or precocious puberty, among girls born between the 1st and 33th percentile (group 1; *n* = 33), the 33th and the 66th percentile (group 2; *n* = 22), and above the 66th percentile (group 3; *n* = 14) of birthweight

	Group 1	Group 2	Group 3	*p* value	Total
Ovary volume (cm^3^)	0.948 0.885–1.337	0.628 0.287–1.031	1.786 0.959–2.910	0.034	0.844 0.537–1.489
Uterus volume (cm^3^)	1.227 0.774–2.004	1.445 0.798–2.669	2.629 1.815–7.678	0.014	1.450 0.831–2.574
Week of birth	39 27–41	39 37.5–40.5	39 37.5–40.5	0.999	39 37.75–40.25
Prem. telarche	34.1%	52.9%	38.6%	0.182	40.1%
Prem. pubarche	26.8%	23.5%	32.8%	0.453	26.0%
Precoc puberty	39.0%	23.5%	28.5%	0.246	33.8%

Ovarian volume was related to girls’ age, expressed in months (*p* = 0.034). When corrected for age, ovarian volume was related to percentiles of birthweight (*p* = 0.034) and to the presence of ovarian follicles (*p* = 0.027) (Table 3). There was no relation of ovarian volume with precocious puberty, (*R*^2^ 0.016; *p* = 0.309) or premature pubarche (*R*^2^ 0.027; *p* = 0.188) vs. premature thelarche**.** In multiple regression analysis (*R*^2^ 0.210; *p* = 0.001), factors significantly related to ovarian volume were percentiles of birthweight (beta coefficient 0.012; 95% CI 0.002; 0.021; *p* = 0.021) and presence of follicles (*p* = 0.015) (Table 3)*.* In an additional analysis, percentiles were substituted by tertiles of percentiles, the first tertile representing the reference and the second and the third tertile being entered as dummy variables. Ovarian volume was significantly related (*R*^2^ 0.237; *p* < 0.001) to the presence of follicles (beta coefficient 0.714; 95% CI 0.174, 1.254; *p* = 0.010) and the third tertile of birthweight percentiles (beta coefficient 1.037; 95% CI 0.291, 1.784; *p* = 0.007).

**Table 3:** Simple and multiple regression models on ovarian or uterine volume (cm^3^) and related factors. Data are reported as coefficient of regression (CR) and its 95% confidence interval (CI)

	Crude beta coefficient (95% CI)	*R*^2^; *p*	Adjusted beta coefficient	*p* value
Ovary volume*				*R*^2^ = 0.210 *p* = 0.001
Ovarian follicles	0.636 (0.073; 1.203)	0.147; 0.027	0.685 (0.139; 1.232)	0.015
Birth weight percentiles	0.011 (0.001; 0.021)	0.134; 0.039	0.012 (0.002; 0.021)	0.021
Uterus volume				*R*^2^ = 0.237 *p* = 0.005
Ovary volume	1.353 (0.335; 2.371)	0.099; 0.010	NS	NS
Birthweight percentiles	0.062 (0.018; 0.106)	0.112; 0.0061	0.067 (0.021; 0.114)	0.005

*Data are corrected for girls’ age expressed in months

Uterine volume was not related to girls’ age. Uterine volume was related to ovarian volume and birthweight percentile (Table 3). There was no relation with premature pubarche (*R*^2^ 0.021; *p* = 0.258) while that with precocious puberty was close to significance (*R*^2^ 0.235; *p* = 0.066). In multiple regression analysis, only percentiles of birthweight remained independently related to uterine volume (beta coefficient 0.067; 95% CI 0.021, 0.114; *R*^2^ 0.237; *p* = 0.005) (Table 3). The result was similar even when precocious puberty was entered into the model.

## Discussion

The present results indicate that in young premenarchal girls with precocious puberty, premature thelarche, or premature pubarche, the volume of ovary and uterus is related to birthweight, a higher birthweight being associated with a larger ovary and uterus.

Prior to menarche, ovarian volume is less than 2 cm^3^ and relatively stable up to puberty [17]. Ovarian volume is related to the amounts of primordial follicles [18, 19]. The amounts of primordial follicles reach peak values at approximately 20 weeks of gestation then it declines to about 1 million at time of birth [20]. The herein reported relation between ovarian volume and birthweight seems to indicate that the amounts of primordial follicles within the ovary depend on intrauterine fetal growth. Interestingly, length of gestation did not impact on uterine or ovarian volume, as to indicate that is the growth of the fetus more than the duration of the pregnancy that exert an influence on the development of reproductive organs.

In our data, uterine volume is linearly related to birthweight. Whether this effect somewhat persists after menarche, it may have an impact on future reproduction, a smaller uterus possibly exerting a negative effect on fetal growth and pregnancy outcome [21, 22].

In a previous prospective study, intrauterine fetal growth, or birthweight corrected for maternal age, parity, or week of gestation, was not related to uterine and ovarian volume of post-menarchal girls (13). That study was aimed to evaluate how maternal factors can influence intrauterine fetal growth and uterus and ovarian volume. In addition, uterus and ovary were evaluated during their post-menarche growth, with a correction that was limited only to years after menarche [13]. The possibility that the relation between birthweight and uterine and ovarian volume is lost after menarche cannot be excluded. However, a lower uterine and ovarian volume was previously reported in post-pubertal girls born SGA [12]. Our results expand those data by showing that a linear relation exists between birthweight and ovary or uterus volume. Likely, in the previous study, SGA were compared with females born in the highest birthweight percentiles, such as those above the 66th percentile of our study [12].

The possibility that intrauterine fetal growth influences future reproductive life needs to be further explored, but it seems to be confirmed by evidence that environmental factors, such as seasons, modulate birthweight and concomitantly influence reproductive capacities and ovarian exhaustion [23]. A reduced fertility was reported also in females born SGA [7], although the data were not confirmed in another investigation [24]. The design of the study did not allow us to evaluate the role played by constitutional, environmental, and maternal factor in determining neonatal birthweight and in particular uterus and ovary volume. Yet the data seems to indicate that whatever factors exert an influence, this influence is concomitantly exerted on body weight and volume of uterus and ovary.

The number of women included in the present study, even though limited, is greater than that of other studies published on this issue [10, 12]. The study was not performed in the general population, but in a subset of premenarchal young females that were evaluated for a suspect of precocious puberty, premature pubarche, or thelarche. Some of these conditions may have an impact on premenarchal ovarian volume [25, 26], although women with premature thelarche or pubarche seem to have ovarian and uterine volume similar to that of normal premenarchal girls [26]. In our analysis, precocious puberty was associated with a tendency to a greater uterine and ovarian volume. In spite of that, birthweight remained an independent determinant of ovary and uterus volume. Some [3–6], but not all [27], the evidence indicates that SGA is associated with an early puberty. In our study, 23 females had been born SGA (< 10 percentile), but the SGA rate was not different among girls with precocious puberty, premature pubarche, or thelarche. We realize that our data were obtained in a particular subset of individuals and that they need to be replicated in more physiological conditions and replicated in girls after menarche.

## Conclusions

Present data expand previous findings obtained in SGA fetuses and indicate that weight at birth is linearly related to the volume of uterus and ovary at 5–9 years of age. This finding needs to be further confirmed in larger and more physiological sets of individuals.

## Data Availability

The datasets used and/or analyzed during the current study are available from the corresponding author on reasonable request.
